# Impact of pharmacist intervention on therapeutic drug monitoring of vancomycin: a meta-analysis

**DOI:** 10.3389/fphar.2026.1805469

**Published:** 2026-04-29

**Authors:** Mubai Ma, Dongnan Cao, Yaqin Pan, Guirong Xiao

**Affiliations:** 1 Department of Pharmacy, West China Hospital, Sichuan University, Chengdu, China; 2 Pharmacy Department, Chengdu Shangjin Nanfu Hospital, Chengdu, China

**Keywords:** acute kidney injury, meta-analysis, pharmacist, therapeutic drug monitoring, vancomycin

## Abstract

**Objective:**

To systematically evaluate the impact of pharmacist interventions on the implementation and clinical efficacy of vancomycin therapeutic drug monitoring (TDM).

**Methods:**

A Cochrane systematic review methodology was employed. Databases including PubMed, Medline, Embase, Cochrane Library, CNKI, and Sinomed were searched. After quality assessment and data extraction of eligible clinical studies, Meta-analysis was performed using Stata 18.0 to compare the differences between the pharmacist intervention group and the non-pharmacist intervention group in terms of the incidence of acute kidney injury, clinical effective rate, 30-day mortality, correct blood sampling time rate, target blood concentration attainment rate, TDM sampling rate, etc.

**Results:**

A total of 63 studies were included in the Meta-analysis. The results showed that pharmacist interventions reduced the incidence of acute kidney injury (RR = 0.67, 95% CI = 0.57, 0.78, P < 0.05), improved the clinical effective rate (RR = 1.11, 95% CI = 1.06, 1.17, P < 0.05), reduced 30-day mortality (RR = 0.52, 95% CI = 0.33, 0.82, P < 0.05), increased the correct TDM blood sampling time rate (RR = 1.52, 95% CI = 1.25, 1.83, P < 0.05), increased the target blood concentration attainment rate (RR = 1.60, 95% CI = 1.49, 1.72, P < 0.05), increased the TDM sampling rate (RR = 1.65, 95% CI = 1.34, 2.04, P < 0.05), increased the proportion of dosage regimen adjustments based on TDM results (RR = 2.68, 95% CI = 1.93, 3.70, P < 0.05). There was no significant effect on the duration of medication, length of hospital stay or TDM timeliness. Subgroup analysis showed that In the 10–20 mg/L group and the group with no specified target range, the serum concentration attainment rate was improved under pharmacist intervention (RR = 1.61, 95% CI = 1.50, 1.73, P < 0.05; RR = 1.49, 95% CI = 1.07, 2.08, P < 0.05). In the groups defined by serum creatinine increase ≥0.5 mg/dL or ≥50% from baseline, and serum creatinine increase ≥0.3 mg/dL or ≥50% from baseline, pharmacist interventions reduced the incidence of acute kidney injury (RR = 0.58, 95% CI = 0.45, 0.74, P < 0.05; RR = 0.64, 95% CI = 0.48, 0.85, P < 0.05).

**Conclusion:**

This study indicates that in patients treated with vancomycin under TDM, pharmacist interventions can promote the standardization of vancomycin TDM, reduce the incidence of acute kidney injury, and improve clinical outcomes.

**Systematic Review Registration:**

https://www.crd.york.ac.uk/PROSPERO/view/CRD420261301721, identifier CRD420261301721.

## 1 Introduction

Therapeutic Drug Monitoring (TDM) involves tailoring individualized dosage regimens for patients by measuring *in vivo* drug exposure, pharmacological markers, or efficacy indicators. Leveraging quantitative pharmacology models and guided by the drug’s therapeutic window, this practice ensures optimal medication outcomes (Zhang et al., 2019). TDM comprises three key components: sample analysis, quantitative calculation, and clinical intervention (Zhang et al., 2019). Accurate sampling timing and methodologies are critical to upholding the scientific validity and reliability of test results (Zhu et al., 2014), while rigorous quantitative analysis, sound result interpretation, and subsequent clinical medication interventions constitute the core work and inherent value of TDM (Zhang et al., 2019).

Since its introduction to the market in 1958, vancomycin has emerged as one of the first-line antimicrobial agents for the management of infections caused by methicillin-resistant *Staphylococcus aureus* (MRSA). Given its well-documented adverse effect profile, most notably nephrotoxicity, clinical practice guidelines universally recommend that vancomycin therapy be guided by therapeutic drug monitoring (TDM) (Filippone et al., 2017; He et al., 2020; Rybak et al., 2020; Matsumoto et al., 2022), with a growing emphasis on the active involvement of clinical pharmacists in delivering TDM services (He et al., 2020; Rybak et al., 2020). In 2000, the Practice Guidelines for Pharmacotherapy Specialists issued by the American College of Clinical Pharmacy first called for pharmacotherapy specialists to participate in therapeutic drug monitoring (TDM): assisting with specimen collection, interpreting reports, providing dosage adjustment recommendations to physicians, and monitoring the recommended regimens (The ACCP Clinical Practice Affairs Committee, Subcommittee B, 1998-1999, 2000). In 2015, China’s Guidelines for Therapeutic Drug Monitoring of Vancomycin recommended the individual calculation and adjustment of vancomycin dosage based on population pharmacokinetics (Ye et al., 2016). In 2020, the U.S. guidelines recommended the Bayesian approach to integrate AUC monitoring results, for which specialized pharmacists are required to participate (Rybak et al., 2020). TDM-based pharmaceutical care includes evaluating vancomycin treatment indications, calculating initial and maintenance dosages, performing ongoing therapeutic monitoring, and providing individualized dose-adjustment recommendations based on TDM results and clinical response (Cardile et al., 2015; Chen et al., 2018; Chen, 2021; Han, 2022).

Pharmacists can improve patient outcomes by targeting key nodes throughout the entire course of vancomycin therapy. Given that the efficacy and toxicity of vancomycin are reliant on TDM, clinical pitfalls such as inappropriate timing of trough concentration sampling and dosing errors (Koppula et al., 2015; Alzahrani et al., 2021; Van Der Heggen et al., 2021) directly lead to biased interpretation of drug concentration data. By educating clinical staff on standardized blood collection protocols and conducting medication order verification, pharmacists ensure accurate timing of trough level sampling. This eliminates erroneous clinical decisions stemming from preanalytical sample errors at the source, laying a reliable foundation for individualized dosing regimens (Xiao et al., 2021; Alrasheed et al., 2025; Yoshikawa et al., 2025). Additionally, pharmacists actively follow up on laboratory test results and maintain real-time communication with the clinical care team (Cheng et al., 2019; Zhang, 2023), which shortens the turnaround time from identifying abnormal drug concentrations to implementing regimen adjustments. This prompt intervention helps patients achieve target drug exposure rapidly, mitigating risks including inadequate infection control and cumulative renal injury associated with delayed therapeutic modifications. Inadequate or excessive vancomycin dosing, along with failure to individualize dosages based on renal function, represents the primary driver of vancomycin treatment failure and heightened toxicity. Guided by pharmacokinetic/pharmacodynamic principles, dynamic changes in renal function, and target trough concentrations or AUC/MIC thresholds, pharmacists formulate individualized dosing strategies and conduct ongoing reassessments (Hirano et al., 2016; Han et al., 2017; Lu et al., 2022). This approach circumvents subtherapeutic or supratherapeutic drug exposure caused by empirical fixed dosing, ultimately enhancing clinical cure rates while reducing the incidence of vancomycin-induced nephrotoxicity.

Existing primary studies on pharmacist-led vancomycin interventions report inconsistent findings, with conflicting results regarding nephrotoxicity risk, clinical cure rates, and target concentration attainment. Furthermore, outcome measures across studies are fragmented: most trials focus on isolated process indicators rather than patient-centered clinical endpoints, and no prior meta-analysis has provided pooled effect estimates to quantify the true clinical impact of pharmaceutical care. Therefore, to investigate whether pharmaceutical care improves the standardization of TDM, as well as the safety and effectiveness of clinical medication, this study retrieved relevant literature on clinical interventions involving pharmacists in the implementation of vancomycin TDM and conducted a meta-analysis to evaluate its impact on clinical outcomes.

## 2 Methods

This meta-analysis were carried out in compliance with the PRISMA 2020 guidelines (Page et al., 2021). The study did not call for ethical clearance or permission because it was a meta-analysis. The protocol for this systematic review was registered retrospectively on PROSPERO (CRD420261301721) after data synthesis. The predefined study protocol was strictly followed without any post-hoc changes to the research plan.

### 2.1 Sources of data and the method of search

A comprehensive literature search was conducted across multiple databases, including PubMed, Medline, Embase, Cochrane Library, CNKI, Sinomed, WanFang, and VIP. The search timeframe was from the inception of each database to 31 August 2025. The search keywords included “vancomycin”, “drug monitoring”, “pharmaceutical care”, and “precision medicine”. The complete search strategy, including specific search terms and combinations, is detailed in Supplementary Material 1. Additionally, this study screened the references of eligible studies and relevant systematic reviews published in the past 5 years to ensure the comprehensiveness of the search.

Two reviewers independently screened and evaluated the titles, abstracts, and full texts of the literature according to the pre-established inclusion and exclusion criteria. Disagreements between the reviewers were resolved through discussion, and a third senior reviewer was consulted for adjudication if necessary to ensure the rigor of the research methodology.

### 2.2 Selection of studies

Studies were considered if they matched the following requirements: (1) Study population: Patients who received intravenous vancomycin and underwent therapeutic drug monitoring after administration. (2) Study Design: The experimental group received pharmacist-led interventions, while the control group had no pharmacist involvement. Pharmacist intervention strategies encompassed: participating in the development of initial medication regimens, advising physicians on TDM implementation, assisting with dose adjustments for patients with subtherapeutic blood concentrations, conducting ongoing medication monitoring, providing specialized TDM consultation services, and overseeing both dosing regimens and the entire TDM process. (3) Outcome Measures: Primary outcomes included the incidence of acute kidney injury, clinical effective rate, and 30-day mortality. Secondary outcomes consisted of the target serum concentration attainment rate, the proportion of dosage adjustments guided by TDM results, TDM sampling rate, correct blood sampling timing rate (Steady-state trough concentrations were obtained, and blood samples were collected within 30 min before the next scheduled dose), TDM timeliness (defined as the number of days between initial vancomycin administration and collection of the first TDM sample), the duration of vancomycin treatment, and hospital length of stay. (4) Study Types: Randomized controlled trials (RCTs) and non-randomized studies (NRS). Non-randomized studies included both prospective and retrospective cohort studies.

Articles were omitted if: (1) Insufficient data to assess the study population and trial design; (2) Duplicate publications (the earliest published version was selected).

### 2.3 Extraction of data

Studies meeting the inclusion criteria were managed using EndNote X9 software to avoid duplicate literature. Two independent reviewers extracted the study data, including basic study information (author, title, year of publication) and outcome measures (as detailed in Table 1).

**TABLE 1:** The basic characteristics of the included studies in the review.

Study	Study design	Country	Subjects (intervention/control)	Outcomes
Chen et al. (2015)	Retrospective	China	184 (90/94)	1,2,7
Chen (2021)	Retrospective	China	75 (41/34)	1,4,5,6,7,
Chen et al. (2018)	Retrospective	China	102 (64/38)	2,5,8,10
Cheng et al. (2019)	Retrospective	China	24 (11/13)	1
Han (2022)	Retrospective	China	86 (40/46)	5,6
Hu et al. (2012)	Retrospective	China	72 (36/36)	1
Jiang (2022)	Prospective	China	66 (33/33)	6,7,9,10
Li (2016)	Retrospective	China	130 (71/59)	5
Liu et al. (2021)	Retrospective	China	179 (63/116)	5
Pan (2021)	Retrospective	China	52 (26/26)	7
Xiao et al. (2021)	Prospective	China	121 (59/62)	3,5,6,7,10
Zhang (2023)	Retrospective	China	188 (113/75)	6,7
Gao et al. (2024)	Retrospective	China	123 (62/61)	5,6,7
Gao et al. (2019)	Retrospective	China	99 (56/43)	3,5
Liao and Liu (2022)	Retrospective	China	172 (86/86)	5,8
Peng et al. (2023)	Retrospective	China	341 (197/144)	5,6
Tao et al. (2020)	Retrospective	China	471 (224/247)	1,2,5,6,7
Xu et al. (2018)	Retrospective	China	258 (100/158)	3,4,5,6,8,9
Cardile et al. (2015)	Retrospective	USA	340 (173/167)	3,5,6
Levin et al. (2016)	Retrospective	USA	183 (108/75)	5
Peyko and Friedman-Jakubovics (2018)	Retrospective	USA	427 (199/228)	1,3
Smith et al. (2016)	Retrospective	USA	198 (149/49)	3,5,6
Brennan et al. (2015)	Retrospective	USA	372 (193/179)	5
Cesario and Kertland (2024)	Retrospective	Canada	161 (89/72)	1
Gitman et al. (2012)	Retrospective	USA	207 (107/100)	5
Harrison et al. (2017)	Retrospective	USA	319 (163/156)	5
Kaplun et al. (2018)	Prospective	USA	226 (84/142)	5
Ma et al. (2019)	RCT	China	152 (72/80)	6
Marquis et al. (2015)	Retrospective	USA	319 (158/161)	6,10
Masoumi et al. (2017)	Retrospective	Iran	200 (100/100)	1,5,6
Momattin et al. (2015)	Retrospective	Saudi Arabia	564 (286/278)	5,6
Olson et al. (2016)	Retrospective	USA	1,629 (777/852)	6
Phillips et al. (2013)	Retrospective	Australia	40 (20/20)	5,6
Robinson et al. (2016)	Retrospective	USA	105 (42/63)	3,5,6
Shankar and Cies (2009)	Retrospective	USA	67 (35/32)	5
Shuler et al. (2021)	Retrospective	USA	200 (100/100)	5,6
Willis et al. (2017)	Retrospective	USA	300 (150/150)	6,7,8
Jiang (2020)	RCT	China	80 (40/40)	7,10
Fang (2017)	RCT	China	137 (72/65)	5,7,8,10
Chen et al. (2022)	Retrospective	China	245 (155/90)	5
Han et al. (2017)	Retrospective	USA	249 (123/126)	5
Hirano et al. (2016)	Retrospective	Japan	79 (51/28)	2,5,6,8,9,10
Imaura et al. (2011)	Retrospective	Japan	58 (11/47)	1,5,9,10
Joseph et al. (2021)	Retrospective	USA	214 (104/110)	1,3,5,6
Komoto et al. (2018)	Retrospective	Japan	77 (28/49)	5
Lu et al. (2022)	Retrospective and prospective	China	374 (138/236)	1,3,4,5,6,7,8
Masuda et al. (2015)	Retrospective	Japan	610 (102/508)	5,6
Maulina et al. (2022)	Retrospective	Latvia	170 (44/126)	5
Maxson et al. (2016)	Retrospective	USA	145 (51/94)	5
Nakashima et al. (2018)	Retrospective	Japan	152 (67/85)	5,6
Phillips and Gordon (2015)	Retrospective	Australia	99 (46/53)	3,5,6
Shao (2019)	Retrospective	China	321 (183/138)	1,6,7
Yang et al. (2021)	Retrospective	China	85 (45/40)	5,7,10
Adamsick et al. (2019)	Retrospective	USA	200 (100/100)	5,6
Al-Ruwaisan et al. (2020)	Retrospective	Saudi Arabia	100 (47/53)	3,6
Chanas et al. (2020)	Retrospective	USA	1,011 (497/514)	5,10
Hanretty et al. (2016)	Retrospective	USA	310 (149/161)	5
Levin and Kiser (2010)	Retrospective	USA	133 (68/65)	5
Sussman and Farkas (2013)	Retrospective	USA	200 (100/100)	5
Welty and Copa (1994)	Prospective	USA	116 (61/55)	1,6,9
Lu (2019)	Retrospective	China	310 (120/190)	3,
Patel (2013)	Retrospective	USA	465 (301/164)	5,6
Okada et al. (2016)	Retrospective	Japan	145 (74/71)	5,6

1: TDM sampling rate, 2: TDM timelinessm, 3: Correct rate of blood sampling time, 4: Proportion of dosage regimen adjustments based on TDM results, 5: Serum concentration attainment rate, 6: Incidence of acute kidney injury (AKI), 7: Clinical effective rate, 8: 30-day mortality, 9: Length of hospital stay, 10: Duration of vancomycin treatment.

### 2.4 Bias risk assessment

Two trained researchers independently read the full texts of the included studies. Non-randomized studies were assessed using the Newcastle-Ottawa Scale (NOS), while randomized controlled trials were evaluated using the updated Cochrane Risk of Bias tool (RoB 2) (Sterne et al., 2019). The assessment results from the two researchers were cross-checked. Discrepancies regarding controversial studies were resolved through discussion, with consultation from a third reviewer if necessary.

### 2.5 Data analysis

All statistical analyses were performed using Stata 18.0. A two-sided significance level of α = 0.05 was adopted. For dichotomous outcomes, pooled effect sizes were estimated using relative risk (RR) or rate difference (RD) with corresponding 95% confidence intervals (CI). For continuous outcomes, weighted mean difference (WMD) with 95% CI was used when outcomes were measured on the same scale; otherwise, standardized mean difference (SMD) with 95% CI was applied. Heterogeneity was evaluated using the Cochran’s χ^2^ (Q) test and the I^2^ statistic. A fixed-effects model (Mantel-Haenszel model) was used if homogeneity was indicated (P > 0.10 and I^2^ ≤ 50%). If significant heterogeneity was detected (P ≤ 0.10 or I^2^ > 50%), a random-effects model (DerSimonian-Laird model) was employed. Egger’s linear regression test and Begg’s rank correlation test were used to evaluate potential publication bias, with P < 0.05 considered indicative of statistically significant publication bias.

## 3 Results

### 3.1 Characteristics of included studies

A total of 1,195 articles were initially retrieved through electronic searches. After removing 795 duplicate records, 400 articles were screened and evaluated at the title and abstract level. Following title and abstract screening, 329 studies were excluded, and the remaining 71 articles were included in the full-text review stage.

**FIGURE 1:**
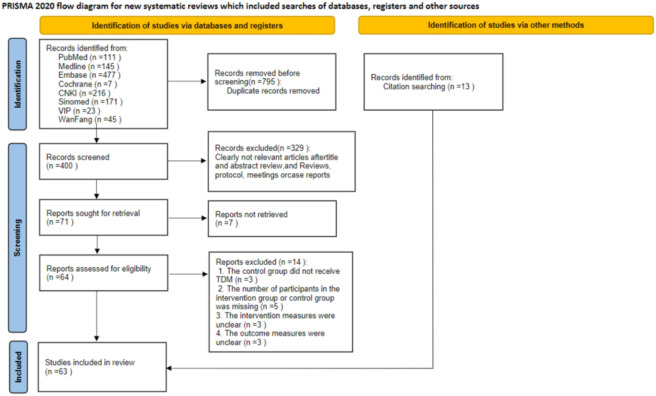
PRISMA Flow diagram of the search process for studies.

Ultimately, this systematic review and meta-analysis included 63 articles, involving a total of 14,837 patients (7,154 in the intervention group and 7,683 in the control group). The included studies comprised 3 randomized controlled trials, 5 prospective cohort studies, and 55 retrospective cohort studies. The screening process for the included studies is shown in Figure 1, and the basic characteristics of the included studies are presented in Table 1.

### 3.2 Safety evaluation results

**FIGURE 2:**
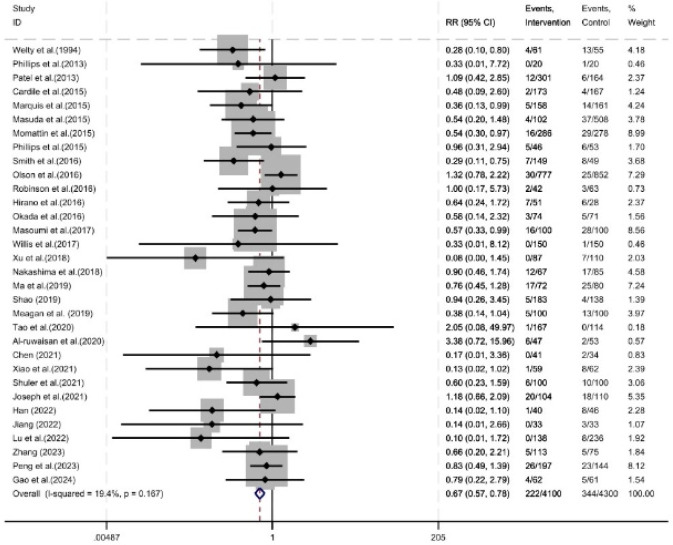
Forest plot of safety evaluation.

As shown in Figure 2, thirty-two studies reported the incidence of acute kidney injury, involving a total of 8,400 patients (4,300 in the control group and 4,100 in the experimental group). Pharmacist intervention reduced the incidence of acute kidney injury (RR = 0.67, 95% CI = 0.57, 0.78, P < 0.05).

**FIGURE 3:**
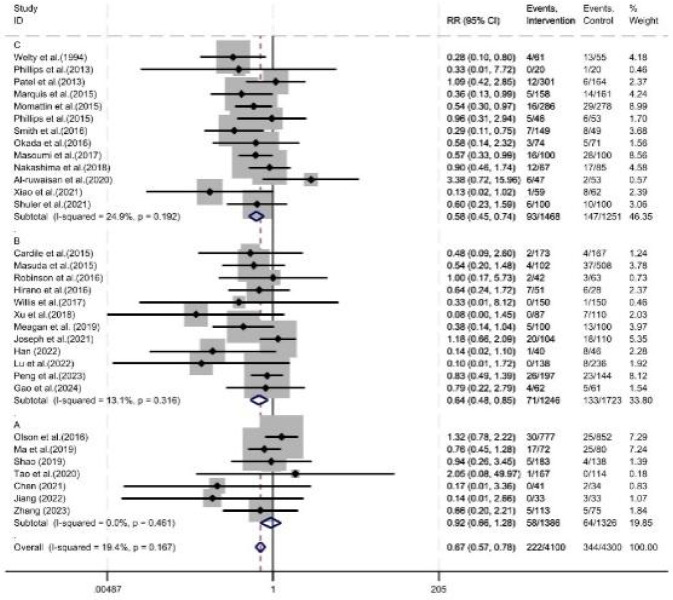
Forest plot of subgroup analysis for safety evaluation. **(A)** Unclear definition of AKI, **(B)** Serum creatinine increased by 0.3 mg/dL or ≥50% from baseline, **(C)** Serum creatinine increased by 0.5 mg/dL or 50% from baseline.

Among the 32 studies that reported the incidence of acute kidney injury, the diagnostic criteria for renal injury varied. Thirteen studies defined acute kidney injury as an increase in serum creatinine of ≥0.5 mg/dL or ≥50% from baseline, 12 studies defined it as an increase in serum creatinine of ≥0.3 mg/dL or ≥50% from baseline, and the remaining 7 studies did not provide a clear definition. Subgroup analysis stratified by definition showed that pharmacist intervention reduced the incidence of acute kidney injury in the group defined by an increase in serum creatinine of ≥0.5 mg/dL or ≥50% from baseline (RR = 0.58, 95% CI = 0.45, 0.74, P < 0.05). In the group defined by an increase in serum creatinine of ≥0.3 mg/dL or ≥50% from baseline, pharmacist intervention was associated with a reduced incidence of acute kidney injury (RR = 0.64, 95% CI = 0.48, 0.85, P < 0.05). In the subgroup with undefined diagnostic criteria, pharmacist intervention showed no significant difference in the incidence of acute kidney injury (RR = 0.92, 95% CI = 0.66, 1.28, P = 0.62) (Figure 3).

### 3.3 Results of efficacy evaluation

#### 3.3.1 Clinical effective rate

**FIGURE 4:**
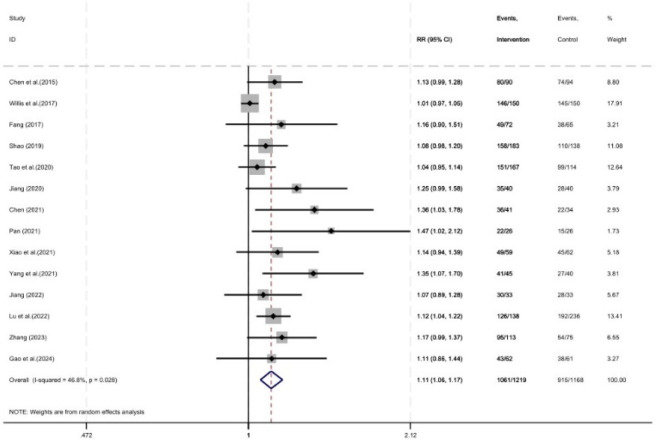
Forest plot of clinical effective rate.

As shown in Figure 4, fourteen studies reported the clinical effective rate, with a total of 2,387 patients included (1,168 in the control group and 1,219 in the intervention group). Pharmacist intervention improved the clinical effective rate of patients (RR = 1.11, 95% CI = 1.06, 1.17, P < 0.05).

#### 3.3.2 30-day mortality

**FIGURE 5:**
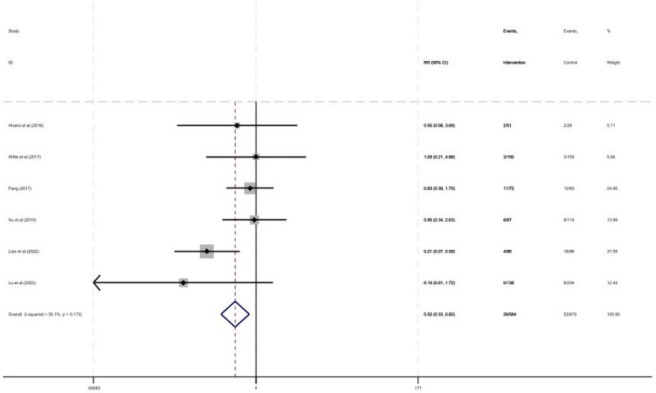
Forest plot of 30-day mortality.

As shown in Figure 5, six studies reported 30-day mortality, with a total of 1,259 patients included (675 in the control group and 584 in the intervention group). Pharmacist intervention reduced 30-day mortality (RR = 0.52, 95% CI = 0.33, 0.82, P < 0.05).

### 3.4 Secondary outcome measures

#### 3.4.1 Correct rate of blood sampling time

**FIGURE 6:**
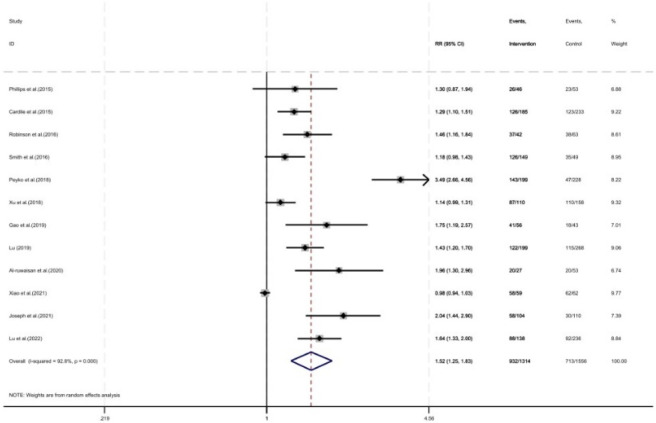
Forest plot of the correct rate of blood sampling time.

As shown in Figure 6, twelve studies reported the correct rate of TDM blood sampling time, with a total of 2,870 patients included (1,556 in the control group and 1,314 in the intervention group). The correct rate of TDM blood sampling time was improved under pharmacist intervention (RR = 1.52, 95% CI = 1.25, 1.83, P < 0.05).

#### 3.4.2 Serum concentration attainment rate

**FIGURE 7:**
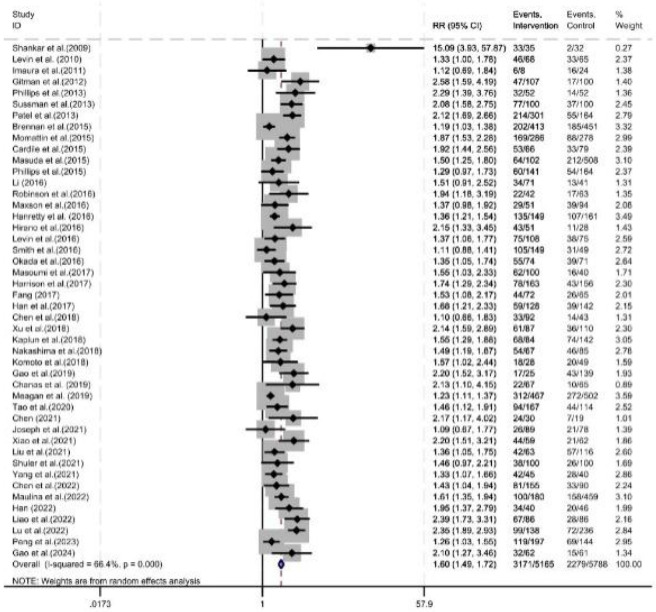
Forest plot of serum concentration attainment rate.

As shown in Figure 7, forty-six studies reported the vancomycin serum concentration attainment rate, with a total of 10,953 patients included (5,788 in the control group and 5,165 in the intervention group). The serum concentration attainment rate was improved under pharmacist intervention (RR = 1.60, 95% CI = 1.49, 1.72, P < 0.05).

**FIGURE 8:**
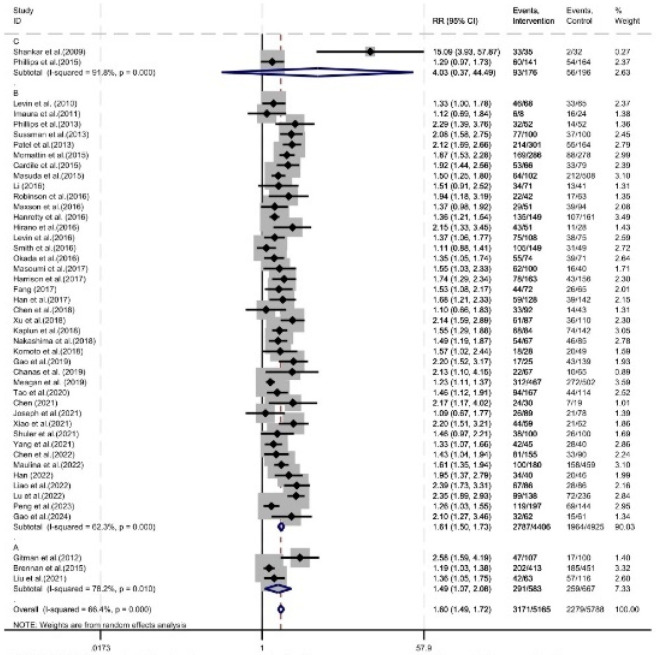
Forest plot of subgroup analysis for serum concentration attainment rate. **(A)** Unclear definition of target trough concentration, **(B)** Target trough concentration range of 10–20 mg/L, **(C)** Target trough concentration range of 15–20 mg/L.

Of the 46 studies reporting the serum concentration attainment rate, 41 studies specified a target trough concentration range of 10–20 mg/L, 2 studies specified a target range of 15–20 mg/L, and the remaining 3 studies did not specify a target trough concentration range. Subgroup analysis according to the target range showed that: In the 10–20 mg/L group and the group with no specified target range, the serum concentration attainment rate was improved under pharmacist intervention (RR = 1.61, 95% CI = 1.50, 1.73, P < 0.05; RR = 1.49, 95% CI = 1.07, 2.08, P < 0.05). In the 15–20 mg/L group, there was no significant difference in the serum concentration attainment rate under pharmacist intervention (RR = 4.03, 95% CI = 0.37, 44.49, P = 0.26) (Figure 8).

#### 3.4.3 TDM sampling rate

**FIGURE 9:**
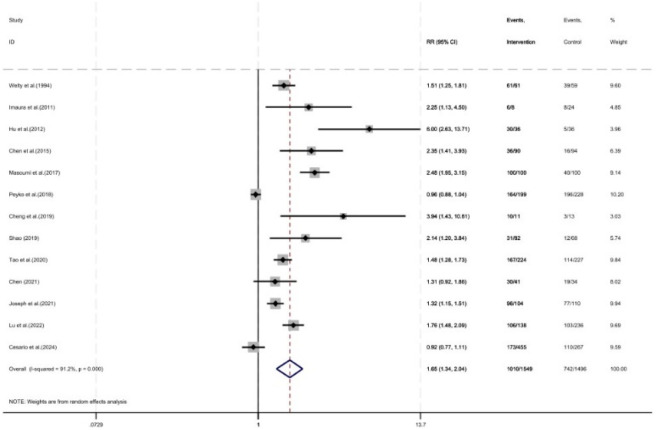
Forest plot of TDM sampling rate.

As shown in Figure 9, thirteen studies reported the vancomycin TDM sampling rate, with a total of 3,045 patients included (1,496 in the control group and 1,549 in the intervention group). The TDM sampling rate was improved under pharmacist intervention (RR = 1.65, 95% CI = 1.34, 2.04, P < 0.05).

#### 3.4.4 Proportion of dosage regimen adjustments based on TDM results

**FIGURE 10:**
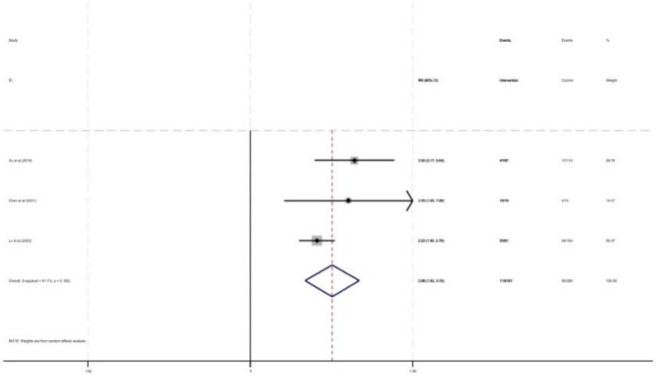
Forest plot of the proportion of dosage regimen adjustments based on TDM results.

As shown in Figure 10, three studies reported the proportion of dosage regimen adjustments based on TDM results, with a total of 455 patients included (288 in the control group and 167 in the intervention group). The proportion of dosage regimen adjustments based on TDM results was increased under pharmacist intervention (RR = 2.68, 95% CI = 1.93, 3.70, P < 0.05).

#### 3.4.5 Duration of vancomycin treatment

**FIGURE 11:**
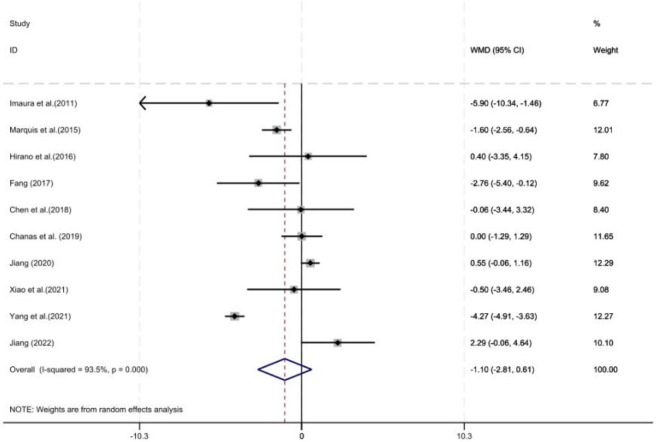
Forest plot of the duration of vancomycin treatment.

As shown in Figure 11, ten studies reported the duration of vancomycin treatment, with a total of 1,198 patients included (547 in the control group and 651 in the intervention group). Pharmacist intervention showed no significant difference in the duration of treatment (WMD = −1.10, 95% CI = −2.81, 0.61, P = 0.21).

#### 3.4.6 Length of hospital stay

**FIGURE 12:**
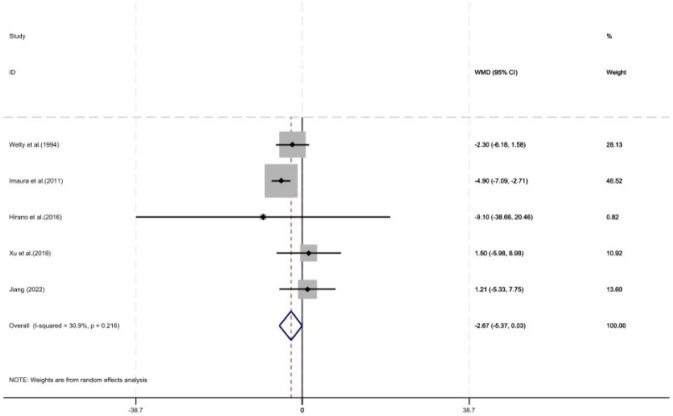
Forest plot of length of hospital stay.

As shown in Figure 12, five studies reported the length of hospital stay, with a total of 577 patients included (321 in the control group and 256 in the intervention group). Pharmacist intervention showed no significant difference in the length of hospital stay (WMD = −2.67, 95% CI = −5.37, 0.03, P = 0.05).

#### 3.4.7 TDM timeliness

**FIGURE 13:**
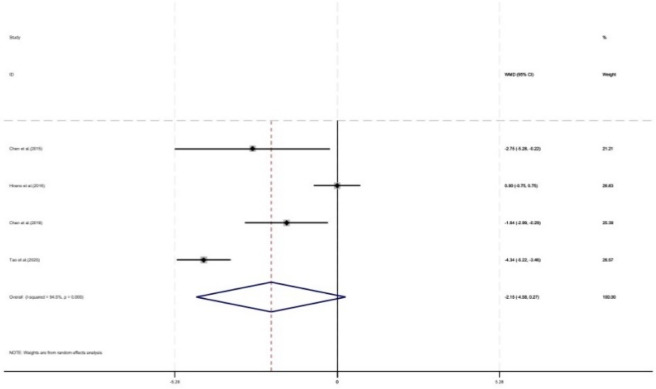
Forest plot of TDM timeliness.

As shown in Figure 13, four studies involving 514 patients (196 in control and 318 in intervention groups) evaluated the impact of pharmacist intervention on TDM timeliness. The result showed that pharmacist intervention tended to improve TDM timeliness (WMD = −2.15, 95% CI: -4.58, 0.27). However, this difference did not reach statistical significance (P = 0.08).

### 3.5 Quality assessment of included studies

All 60 included studies were non-randomized studies (NRS), and their risk of bias was evaluated using the Newcastle-Ottawa Scale (NOS). In contrast, three studies were randomized controlled trials (RCTs), for which the Risk of Bias 2 (RoB 2) tool was employed to assess bias risk. Studies were categorized by quality based on total scores: those with a score of ≥7 points were designated as high quality, 4–6 points as moderate quality, and ≤3 points as low quality.

The quality distribution of the included non-randomized studies was as follows: 39 high-quality studies, 15 moderate-quality studies, and 6 low-quality studies. Notably, the three RCTs included in the analysis were identified to have certain methodological issues. Detailed results of the bias risk assessment and quality classification are presented in Supplementary Material 2.

### 3.6 Risk of bias

Egger’s linear regression and Begg’s rank correlation tests were utilized to assess publication bias across 10 outcome measures. A P-value <0.05 was deemed indicative of significant publication bias, with detailed testing results summarized in Supplementary Material 3. Specifically, publication bias was detected in five outcomes: incidence of acute kidney injury, clinical effective rate, 30-day mortality rate, accuracy rate of blood sampling time, serum concentration attainment rate and TDM sampling rate.

## 4 Discussion

A total of 63 articles were included in this meta-analysis. The findings demonstrated that pharmacist interventions were associated with improved clinical outcomes in terms of safety and efficacy, and were associated with reductions in the incidence of acute kidney injury and 30-day mortality. Specifically, pharmacist involvement markedly improved key TDM-related metrics: the correct timing rate of vancomycin blood sampling for TDM, target blood concentration attainment rate, TDM testing rate, and the proportion of dosage regimen adjustments guided by TDM results. Notably, pharmacist interventions exerted no significant impact on either treatment duration, hospital length of stay or TDM timeliness.

A systematic review examined the association between pharmacist interventions and vancomycin-induced nephrotoxicity. Kunming et al. (2023) observed that pharmacist interventions can mitigate vancomycin-associated acute kidney injury (AKI), presumably by enhancing the rational use of vancomycin, standardizing vancomycin blood concentration monitoring, and closely surveilling patients’ renal function. Notably, the included studies employed inconsistent definitions of AKI. Currently, multiple international classification criteria for AKI exist, including the definition of vancomycin-induced nephrotoxicity outlined in the 2009 consensus statement and review on vancomycin therapeutic monitoring in adult patients—jointly published by the American Society of Health-System Pharmacists (ASHP), the Infectious Diseases Society of America (IDSA), and the Society of Infectious Diseases Pharmacists (SIDP) (Rybak et al., 2009), —as well as the RIFLE, AKIN, and KDIGO criteria (Kellum et al., 2021), all of which differ in their specifications for serum creatinine thresholds.

For this safety endpoint, we stratified the included literature into three subgroups based on AKI definitions. The findings revealed that pharmacist interventions reduced AKI incidence in the two subgroups utilizing clear, standardized definitions; in contrast, no significant difference in AKI incidence was observed with pharmacist intervention in the subgroup with ambiguous AKI definitions. This between-subgroup discrepancy is unlikely to reflect ineffectiveness of pharmacist interventions in the latter subgroup, but rather more likely stems from methodological bias. Studies with unclear AKI definitions may have failed to implement standardized laboratory monitoring protocols, contained missing retrospective data, or relied on subjective judgment criteria—all factors that could obscure the true effect of the interventions. Conversely, this result underscores the critical necessity of adopting standardized definitions in both clinical research and practice, as such standardization serves as the foundation for ensuring the comparability, interpretability, and clinical generalizability of research findings. This also highlights that when implementing vancomycin management programs, healthcare institutions should not only promote the proactive integration of pharmacists into the care team but also concurrently standardize the monitoring and diagnostic criteria for AKI. Doing so is essential to accurately validate the clinical benefits of such intervention strategies.

Our research confirms that pharmacist interventions were associated with improvements in the accuracy and timeliness of vancomycin TDM, ultimately contributing to a higher target blood concentration attainment rate. Multiple studies have underscored that inappropriate TDM utilization—including incorrect indication selection, non-standardized sampling timelines, and unscientific result interpretation—can diminish TDM effectiveness and lead to substantial healthcare resource waste (Ab Rahman et al., 2013; Dalaklioglu, 2013; Al Za Abi et al., 2015). Standardized sampling serves as a fundamental prerequisite for the effective implementation of TDM. Following medication administration, drug concentrations fluctuate continuously over time; thus, improper sampling timing fails to accurately capture the *in vivo* drug concentration profile, which in turn precipitates erroneous dosage adjustments. As such, ensuring correct blood sampling timing is paramount to TDM success. Notably, in four included studies, the correct blood sampling rate in the non-pharmacist intervention group fell below 50% (Al-ruwaisan et al., 2019; Xiao et al., 2020; Joseph et al., 2021; Lu et al., 2022). This finding underscores that standardizing TDM sampling timing is a critical responsibility of pharmacists. Concurrently, targeted training for physicians and nurses should be expanded to improve the accuracy of blood sampling practices, thereby reinforcing the integrity of the entire TDM process.

Currently, trough concentration remains a reliable predictor of vancomycin efficacy and safety, with steady-state trough concentrations (10–20 mg/L) recommended as the standard target for vancomycin monitoring (Rybak et al., 2009; Matsumoto et al., 2013). It is worth noting that while our analysis included studies predominantly employing trough-based monitoring (target range 10–20 mg/L), contemporary international guidelines have increasingly endorsed area under the curve AUC/MIC-based monitoring strategies to optimize vancomycin efficacy while minimizing nephrotoxicity (Rybak et al., 2020). This shift reflects a growing recognition that AUC-based dosing better accounts for individual pharmacokinetic variability. Notably, the included studies exhibited inconsistencies in their specifications for vancomycin’s target trough concentration range. To address this variability, we stratified the included literature into three subgroups based on the specified target values. The findings revealed that the vast majority of studies adopted the most widely used target range in clinical practice (10–20 mg/L). Within this range, pharmacist interventions were associated with an improvement in the blood concentration attainment rate—a finding consistent with our *a priori* expectations. In contrast, no significant advantage of pharmacist interventions was observed in studies employing a narrower target range (15–20 mg/L). We hypothesize that, in the non-pharmacist intervention group, physicians may have adjusted dosages more aggressively due to the stricter nature of this narrower target range. This heightened clinical vigilance likely narrowed the “room for improvement” between the non-pharmacist and pharmacist intervention groups. Additionally, only two articles were included in this latter subgroup, highlighting the need for larger sample sizes in future analyses to validate these findings.

A key strength of this study lies in its employment of a comprehensive search strategy across multiple databases, coupled with the inclusion of relevant literature from both domestic and international sources. This approach positions the study as one of the most thorough analyses to date examining the impact of pharmacist interventions on the effectiveness of vancomycin therapeutic drug monitoring (TDM), with outcome measures encompassing both safety endpoints and multiple efficacy indicators.

This study is also subject to several limitations. First, the majority of included studies were non-randomized, which may have introduced baseline imbalances. We additionally conducted a meta-analysis specifically focused on these non-randomized studies, and the findings were not significantly different from those of the overall analysis. Owing to the limited number of available publications, we were unable to analyze the outcome indicators of the three included randomized controlled trials (RCTs). Second, heterogeneity was observed across several outcome indicators. For pooled effect sizes exhibiting high heterogeneity (I^2^ > 50%), this study utilized a random-effects model but did not employ approaches such as meta-regression to investigate the sources of this heterogeneity. Third, publication bias was assessed using Egger’s regression test. Significant publication bias was detected for the outcomes of incidence of acute kidney injury, Clinical effective rate, Accuracy rate of blood sampling time, Serum concentration attainment rate and TDM sampling rate. For AKI incidence, a negative Z value suggested a potential overestimation of nephrotoxicity risk in small studies. For the remaining four outcomes, positive Z values indicated that small studies tended to overestimate the beneficial effects of pharmacist interventions. No significant publication bias was observed for other outcomes, indicating more robust pooled estimates for these endpoints. Second, publication bias is prevalent in studies of pharmacist-led interventions, and its causes can be analyzed from multiple dimensions: First, most pharmacist intervention studies are single-center, small-sample investigations, with non-randomized controlled trials or before-after studies as the dominant design. Such studies exhibit substantial heterogeneity in methodological quality. Second, the implementation of pharmacist interventions is often closely tied to clinical practice changes within healthcare institutions. Intervention programs demonstrating successful outcomes are more likely to be prepared and published, while those failing to show significant effects are less frequently disseminated through academic channels. This bias has important implications for the certainty and strength of the conclusions drawn in this study. Although our analysis demonstrates that pharmacist interventions were associated with improved clinical outcomes, including a reduction in the incidence of acute kidney injury and improvements in clinical effective rates and serum concentration attainment rates, these findings should be interpreted with caution. Publication bias may lead to overestimation of the pooled effect sizes, thereby weakening the robustness of the conclusions to some extent. In particular, subgroup analyses with a smaller number of included studies and greater heterogeneity are more susceptible to bias in their effect size estimates. Fourth, the degree of pharmacist involvement varied substantially across the included studies, meaning the results of this analysis can only reflect the aggregate effect of pharmacist interventions rather than the impact of specific intervention intensities or approaches. Fifth, the study’s inclusion criteria were not restricted to patients with a specific disease type. As such, further research is urgently warranted to explore whether pharmacist-led TDM interventions confer greater benefits for particular patient populations.

## 5 Conclusion

This systematic review demonstrates that pharmacist interventions positively optimize vancomycin TDM processes and improve patients’ clinical outcomes. Clinically, pharmacist involvement reduces acute kidney injury incidence and 30-day mortality while enhancing clinical response rates. For TDM implementation quality, interventions significantly increase TDM testing rates, correct blood sampling timing, target blood concentration attainment, and the proportion of dosage adjustments guided by TDM results. Notably, pharmacist interventions did not significantly impact hospital length of stay, treatment duration and TDM timeliness.

Subgroup analysis further showed that pharmacist-driven improvements in blood concentration attainment were most pronounced in studies using the 10–20 mg/L target range; pharmacist interventions also more effectively reduced acute kidney injury when defined by international standards. This highlights that unified therapeutic targets and standardized outcome definitions are critical prerequisites for accurately assessing and realizing the value of pharmacist interventions.

In conclusion, integrating pharmacists into vancomycin treatment teams and implementing structured TDM and dosage optimization effectively improves treatment quality and patient safety. We recommend promoting pharmacist-led vancomycin management programs in clinical practice, alongside standardized therapeutic targets and adverse reaction evaluation criteria, to maximize clinical benefits.

## Data Availability

The original contributions presented in the study are included in the article/Supplementary Material, further inquiries can be directed to the corresponding author.
