# Supplementary material for: Impact of pharmacist intervention on therapeutic drug monitoring of vancomycin: a meta-analysis
Source: Front Pharmacol. 2026 Apr 29;17:1805469. doi: 10.3389/fphar.2026.1805469 (PMC13167942; doi:10.3389/fphar.2026.1805469)
Supplement: Supplementary file 1 [file Supplementaryfile1.docx]

**Supplementary**

**Supplementary 1: Search Strategy**

***Search Strategy (PubMed):***

| 41 | ((((vancomycin*) AND ((((((((((("Drug Monitoring"[Mesh]) OR (Monitoring, Drug)) OR (Therapeutic drug monitoring)) OR (TDM)) OR (pharmacokinetic service)) OR (serum concentration)) OR (blood concentration)) OR (plasma concentration)) OR (drug concentration)) OR (trough concentration)) OR (trough level))) AND ((concentration) OR (trough))) AND (pharmacist*)) AND ((((((((((((((((((((((dosage adjustment) OR (dose adjustment)) OR (adjust,dosage)) OR (adjust,dose)) OR (dose calculation)) OR (dose computation)) OR (calculate, dose)) OR (dosage optimization)) OR (pharmacist intervention)) OR ("pharmaceutical services"[Mesh])) OR (pharmac* service*)) OR (service*,pharmac*)) OR (pharmaceutical care)) OR (pharmacy practice)) OR (practice pattern*, pharmacy)) OR (clinical pharmacy)) OR ("precision medicine"[Mesh])) OR (personali* medicine)) OR (personali* therapy)) OR (individuali* medicine)) OR (individuali* therapy)) OR (individual* administ*)) |
| --- | --- |
| 40 | (((((((((((((((((((((dosage adjustment) OR (dose adjustment)) OR (adjust,dosage)) OR (adjust,dose)) OR (dose calculation)) OR (dose computation)) OR (calculate, dose)) OR (dosage optimization)) OR (pharmacist intervention)) OR ("pharmaceutical services"[Mesh])) OR (pharmac* service*)) OR (service*,pharmac*)) OR (pharmaceutical care)) OR (pharmacy practice)) OR (practice pattern*, pharmacy)) OR (clinical pharmacy)) OR ("precision medicine"[Mesh])) OR (personali* medicine)) OR (personali* therapy)) OR (individuali* medicine)) OR (individuali* therapy)) OR (individual* administ*) |
| 39 | individual* administ* |
| 38 | individuali* therapy |
| 37 | individuali* medicine |
| 36 | personali* therapy |
| 35 | personali* medicine |
| 34 | "precision medicine"[Mesh] |
| 33 | clinical pharmacy |
| 32 | practice pattern*, pharmacy |
| 31 | pharmacy practice |
| 30 | pharmaceutical care |
| 29 | service*,pharmac* |
| 28 | pharmac* service* |
| 27 | "pharmaceutical services"[Mesh] |
| 26 | pharmacist intervention |
| 25 | dosage optimization |
| 24 | calculate, dose |
| 23 | dose computation |
| 22 | dose calculation |
| 21 | adjust,dose |
| 20 | adjust,dosage |
| 19 | dose adjustment |
| 18 | dosage adjustment |
| 17 | pharmacist* |
| 16 | (concentration) OR (trough) |
| 15 | trough |
| 14 | concentration |
| 13 | (((((((((("Drug Monitoring"[Mesh]) OR (Monitoring, Drug)) OR (Therapeutic drug monitoring)) OR (TDM)) OR (pharmacokinetic service)) OR (serum concentration)) OR (blood concentration)) OR (plasma concentration)) OR (drug concentration)) OR (trough concentration)) OR (trough level) |
| 12 | trough level |
| 11 | trough concentration |
| 10 | drug concentration |
| 9 | plasma concentration |
| 8 | blood concentration |
| 7 | serum concentration |
| 6 | pharmacokinetic service |
| 5 | TDM |
| 4 | Therapeutic drug monitoring |
| 3 | Monitoring, Drug |
| 2 | "Drug Monitoring"[Mesh] |
| 1 | vancomycin* |

***Search Strategy (MEDLINE):***

| 1 | vancomycin*.af. |
| --- | --- |
| 2 | 'drug monitoring'.af. |
| 3 | ((monitoring, and drug) or (therapeutic and drug and monitoring) or tdm or (pharmacokinetic and service) or (serum and concentration) or (blood and concentration) or (plasma and concentration) or (drug and concentration) or (trough and concentration) or (trough and level)).af. |
| 4 | 2 or 3 |
| 5 | (concentration or trough).af. |
| 6 | pharmacist*.af. |
| 7 | ((dosage and adjustment) or (dose and adjustment) or adjust,dosage or adjust,dose or (dose and calculation) or (dose and computation) or (calculate, and dose) or (dosage and optimization) or (pharmacist and intervention) or (pharmac* and service*) or service*,pharmac* or (pharmaceutical and care) or (pharmacy and practice) or (practice and pattern*, and pharmacy) or (clinical and pharmacy) or (personali* and medicine) or (personali* and therapy) or (individuali* and medicine) or (individuali* and therapy) or (individual* and administ*) or 'pharmaceutical services' or 'precision medicine').af. |
| 8 | 1 and 4 and 5 and 6 and 7 |

***Search Strategy (Embase):***

| #8 | #1 AND #4 AND #5 AND #6 AND #7 |
| --- | --- |
| #7 | dosage AND adjustment OR (dose AND adjustment) OR adjust,dosage OR adjust,dose OR (dose AND calculation) OR (dose AND computation) OR (calculate, AND dose) OR (dosage AND optimization) OR (pharmacist AND intervention) OR (pharmac* AND service*) OR service*,pharmac* OR (pharmaceutical AND care) OR (pharmacy AND practice) OR (practice AND pattern*, AND pharmacy) OR (clinical AND pharmacy) OR (personali* AND medicine) OR (personali* AND therapy) OR (individuali* AND medicine) OR (individuali* AND therapy) OR (individual* AND administ*) OR 'pharmaceutical services'/exp OR 'precision medicine'/exp |
| #6 | pharmacist* |
| #5 | concentration OR trough |
| #4 | #2 OR #3 |
| #3 | monitoring, AND drug OR (therapeutic AND drug AND monitoring) OR tdm OR (pharmacokinetic AND service) OR (serum AND concentration) OR (blood AND concentration) OR (plasma AND concentration) OR (drug AND concentration) OR (trough AND concentration) OR (trough AND level) |
| #2 | drug monitoring'/exp |
| #1 | vancomycin* |

***Search Strategy (Cochrane):***

| #1 (vancomycin*):ti,ab,kw |
| --- |
| #2 MeSH descriptor: [Drug Monitoring] explode all trees |
| #3 (Monitoring, Drug):ti,ab,kw |
| #4 (Therapeutic drug monitoring):ti,ab,kw |
| #5 (TDM):ti,ab,kw |
| #6 (pharmacokinetic service):ti,ab,kw |
| #7 (serum concentration):ti,ab,kw |
| #8 (blood concentration):ti,ab,kw |
| #9 (plasma concentration):ti,ab,kw |
| #10 (drug concentration):ti,ab,kw |
| #11 (trough concentration):ti,ab,kw |
| #12 (trough level):ti,ab,kw |
| #13 #2 or #3 or #4 or #5 or #6 or #7 or #8 or #9 or #10 or #11 or #12 |
| #14 (concentration):ti,ab,kw |
| #15 (trough):ti,ab,kw |
| #16 #14 or #15 |
| #17 (pharmacist*) |
| #18 (dosage adjustment) |
| #19 (dose adjustment) |
| #20 (adjust,dosage) |
| #21 (adjust,dose) |
| #22 (dose calculation) |
| #23 (dose computation) |
| #24 (calculate, dose) |
| #25 (dosage optimization) |
| #26 (pharmacist intervention) |
| #27 MeSH descriptor: [Pharmaceutical Services] explode all trees |
| #28 (pharmac* service*) |
| #29 (pharmaceutical care) |
| #30 (pharmacy practice) |
| #31 (clinical pharmacy) |
| #32 MeSH descriptor: [Precision Medicine] explode all trees |
| #33 (personali* medicine) |
| #34 (personali* therapy) |
| #35 (individuali* medicine) |
| #36 (individuali* therapy) |
| #37 (individual* administ*) |
| #38 #18 or #19 or #20 or #21 or #22 or #23 or #24 or #25 or #26 or #27 or #28 or #29 or #30 or #31 or #32 or #33 or #34 or #35 or #36 or #37 |
| #39 #1 and #13 and #16 and #17 and #38 |

**Supplementary 2: Quality assessment of included studies**

**Table 2.1** **Non-RCT literature quality evaluation results**

| Study | Select | | | | Comparability | Outcome | | | Total score |
| --- | --- | --- | --- | --- | --- | --- | --- | --- | --- |
|  | A | B | C | D | E | F | G | H |  |
| Chen et al.(2015) | 0 | 1 | 1 | 1 | 2 | 1 | 1 | 1 | 8 |
| Chen (2021) | 0 | 1 | 1 | 1 | 1 | 1 | 1 | 1 | 7 |
| Chen et al.(2018) | 1 | 1 | 1 | 0 | 1 | 1 | 0 | 1 | 6 |
| Cheng et al.(2019) | 0 | 1 | 1 | 1 | 1 | 1 | 1 | 1 | 7 |
| Han (2022) | 1 | 1 | 1 | 1 | 1 | 1 | 1 | 1 | 8 |
| Hu et al.(2012) | 0 | 1 | 1 | 0 | 1 | 1 | 1 | 1 | 6 |
| Jiang (2022) | 0 | 1 | 1 | 1 | 1 | 1 | 1 | 1 | 7 |
| Li (2016) | 1 | 1 | 1 | 1 | 0 | 1 | 1 | 1 | 7 |
| Liu et al.(2021) | 1 | 1 | 1 | 1 | 0 | 1 | 1 | 1 | 7 |
| Pan (2021) | 0 | 1 | 1 | 1 | 0 | 1 | 1 | 1 | 6 |
| Xiao et al.(2021) | 0 | 1 | 1 | 1 | 0 | 0 | 0 | 0 | 3 |
| Zhang (2023) | 1 | 1 | 1 | 1 | 2 | 1 | 1 | 1 | 9 |
| Gao et al.(2024) | 0 | 1 | 1 | 1 | 1 | 1 | 1 | 1 | 7 |
| Gao et al.(2019) | 1 | 0 | 0 | 1 | 0 | 1 | 0 | 1 | 4 |
| Liao et al.(2022) | 1 | 1 | 1 | 1 | 2 | 1 | 1 | 1 | 9 |
| Peng et al.(2023) | 0 | 1 | 1 | 0 | 1 | 1 | 1 | 1 | 6 |
| Tao et al.(2020) | 0 | 1 | 1 | 1 | 0 | 1 | 1 | 1 | 6 |
| Xu et al.(2018) | 1 | 1 | 1 | 0 | 2 | 1 | 1 | 1 | 8 |
| Cardile et al.(2015) | 1 | 1 | 1 | 1 | 1 | 1 | 1 | 1 | 8 |
| Levin et al.(2016) | 0 | 1 | 1 | 0 | 0 | 1 | 1 | 1 | 5 |
| Peyko et al.(2018) | 1 | 1 | 1 | 0 | 0 | 1 | 1 | 1 | 6 |
| Smith et al.(2016) | 1 | 1 | 1 | 0 | 2 | 1 | 0 | 1 | 7 |
| Brennan et al.(2015) | 1 | 1 | 1 | 1 | 0 | 1 | 1 | 1 | 7 |
| Cesario et al.(2024) | 1 | 1 | 1 | 1 | 0 | 1 | 1 | 1 | 7 |
| Gitman et al.(2012) | 1 | 1 | 1 | 1 | 0 | 1 | 1 | 1 | 7 |
| Harrison et al.(2017) | 1 | 1 | 1 | 0 | 2 | 1 | 0 | 1 | 7 |
| Kaplun et al.(2018) | 1 | 1 | 1 | 1 | 2 | 1 | 1 | 1 | 9 |
| Marquis et al.(2015) | 1 | 1 | 1 | 1 | 0 | 1 | 1 | 1 | 7 |
| Masoumi et al.(2017) | 1 | 1 | 0 | 0 | 0 | 0 | 0 | 0 | 2 |
| Momattin et al.(2015) | 1 | 1 | 1 | 0 | 2 | 1 | 0 | 1 | 7 |
| Olson et al.(2016) | 0 | 1 | 1 | 1 | 0 | 1 | 1 | 1 | 6 |
| Phillips et al.(2013) | 1 | 1 | 1 | 1 | 2 | 0 | 1 | 1 | 8 |
| Robinson et al.(2016) | 1 | 1 | 1 | 0 | 0 | 0 | 0 | 0 | 3 |
| Shankar et al.(2009) | 0 | 1 | 1 | 1 | 0 | 1 | 1 | 1 | 6 |
| Shuler et al.(2021) | 1 | 1 | 1 | 0 | 0 | 0 | 0 | 0 | 3 |
| Willis et al.(2017) | 0 | 1 | 1 | 1 | 0 | 1 | 1 | 1 | 6 |
| Chen et al.(2022) | 1 | 1 | 1 | 0 | 2 | 1 | 0 | 1 | 7 |
| Han et al.(2017) | 1 | 1 | 1 | 1 | 0 | 1 | 1 | 1 | 7 |
| Hirano et al.(2016) | 1 | 1 | 1 | 0 | 2 | 1 | 0 | 1 | 7 |
| Imaura et al.(2011) | 1 | 1 | 1 | 0 | 2 | 1 | 0 | 1 | 7 |
| Joseph et al.(2021) | 1 | 1 | 1 | 0 | 1 | 1 | 0 | 1 | 6 |
| Komoto et al.(2018) | 0 | 1 | 1 | 1 | 1 | 1 | 1 | 1 | 7 |
| Lu et al.(2022) | 1 | 1 | 1 | 1 | 2 | 1 | 1 | 1 | 9 |
| Masuda et al.(2015) | 1 | 1 | 1 | 1 | 2 | 1 | 1 | 1 | 9 |
| Maulina et al.(2022) | 1 | 0 | 1 | 1 | 0 | 1 | 1 | 1 | 6 |
| Maxson et al.(2016) | 0 | 1 | 1 | 1 | 1 | 1 | 1 | 1 | 7 |
| Nakashima et al.(2018) | 1 | 1 | 1 | 1 | 2 | 1 | 1 | 1 | 9 |
| Phillips et al.(2015) | 1 | 1 | 1 | 1 | 0 | 1 | 1 | 1 | 7 |
| Shao (2019) | 1 | 1 | 1 | 1 | 1 | 1 | 0 | 1 | 7 |
| Yang et al.( 2021) | 1 | 1 | 1 | 0 | 1 | 1 | 0 | 1 | 6 |
| Meagan et al. (2019) | 1 | 1 | 1 | 0 | 2 | 1 | 0 | 1 | 7 |
| Al-ruwaisan et al.(2020) | 0 | 0 | 1 | 0 | 2 | 0 | 0 | 1 | 3 |
| Chanas et al. (2019) | 1 | 1 | 1 | 0 | 2 | 1 | 0 | 1 | 7 |
| Hanretty et al. (2016) | 0 | 0 | 1 | 0 | 0 | 0 | 0 | 0 | 1 |
| Levin et al. (2010) | 1 | 1 | 1 | 0 | 2 | 1 | 0 | 0 | 6 |
| Sussman et al.( 2013) | 1 | 1 | 1 | 0 | 2 | 1 | 0 | 1 | 7 |
| Welty et al.(1994) | 1 | 1 | 1 | 1 | 2 | 1 | 1 | 1 | 9 |
| Lu (2019) | 1 | 1 | 1 | 1 | 1 | 1 | 1 | 1 | 8 |
| Patel et al.(2013) | 1 | 1 | 1 | 1 | 2 | 1 | 1 | 1 | 9 |
| Okada et al.(2016) | 0 | 1 | 1 | 1 | 2 | 1 | 1 | 1 | 8 |

A：Is the case definition adequate B：Representativeness of the cases C：Ascertainment of exposure D：Demonstration that outcome of interest was not present at start of study E：Comparability of cohorts on the basis of the design or analysis F：Assessment of outcome G：Was follow-up long enough for outcomes to occur H：Adequacy of follow up of cohort

**Table 2.2** **RCT literature quality evaluation results**

| Author | Bias arising from the randomization process | Bias due to deviations from intended intervention | Bias due to missing outcome data | Bias in measurement of the outcome | Bias in selection of the reported result | Overall |
| --- | --- | --- | --- | --- | --- | --- |
| Ma et al.(2019) | Some concerns | Low | Low | Some concerns | Low | Some concerns |
| Jiang (2020) | Some concerns | Low | Low | Some concerns | Low | Some concerns |
| Fang (2017) | Some concerns | Low | Low | Some concerns | Low | Some concerns |

**Supplementary 3:** **Risk of bias**

**Table 3 Risk of bias in review**

| Outcome measures | Number of included studies | Meta bias, Begg | | Meta bias, Egger | |
| --- | --- | --- | --- | --- | --- |
|  |  | Z | P | Z | P |
| Incidence of acute kidney injury | 32 | -1.64 | 0.108 | -2.73 | 0.006 |
| Clinical effective rate | 14 | 1.75 | 0.080 | 4.32 | 0.000 |
| 30-day mortality rate | 6 | -1.88 | 0.133 | -1.01 | 0.312 |
| Accuracy rate of blood sampling time | 12 | 1.44 | 0.150 | 3.04 | 0.002 |
| Serum concentration attainment rate | 46 | 2.05 | 0.041 | 3.43 | 0.001 |
| TDM sampling rate | 13 | 0.92 | 0.360 | 3.77 | 0.000 |
| Proportion of dosage regimen adjustments based on TDM results | 3 | 0.00 | 1.000 | 1.62 | 0.105 |
| Duration of vancomycin treatment | 10 | 0.18 | 0.858 | 0.32 | 0.749 |
| Length of hospital stay | 5 | -0.24 | 1.000 | 0.298 | 0.771 |
| TDM timeliness | 4 | -1.02 | 0.734 | -0.18 | 0.861 |
